# Supplementary material for: Mild Muscle Mitochondrial Fusion Distress Extends Drosophila Lifespan through an Early and Systemic Metabolome Reorganization
Source: Int J Mol Sci. 2021 Nov 9;22(22):12133. doi: 10.3390/ijms222212133 (PMC8618903; doi:10.3390/ijms222212133)
Supplement: Supplementary file 1 [file ijms-22-12133-s001.zip › ijms-1431291-supplementary.pdf]

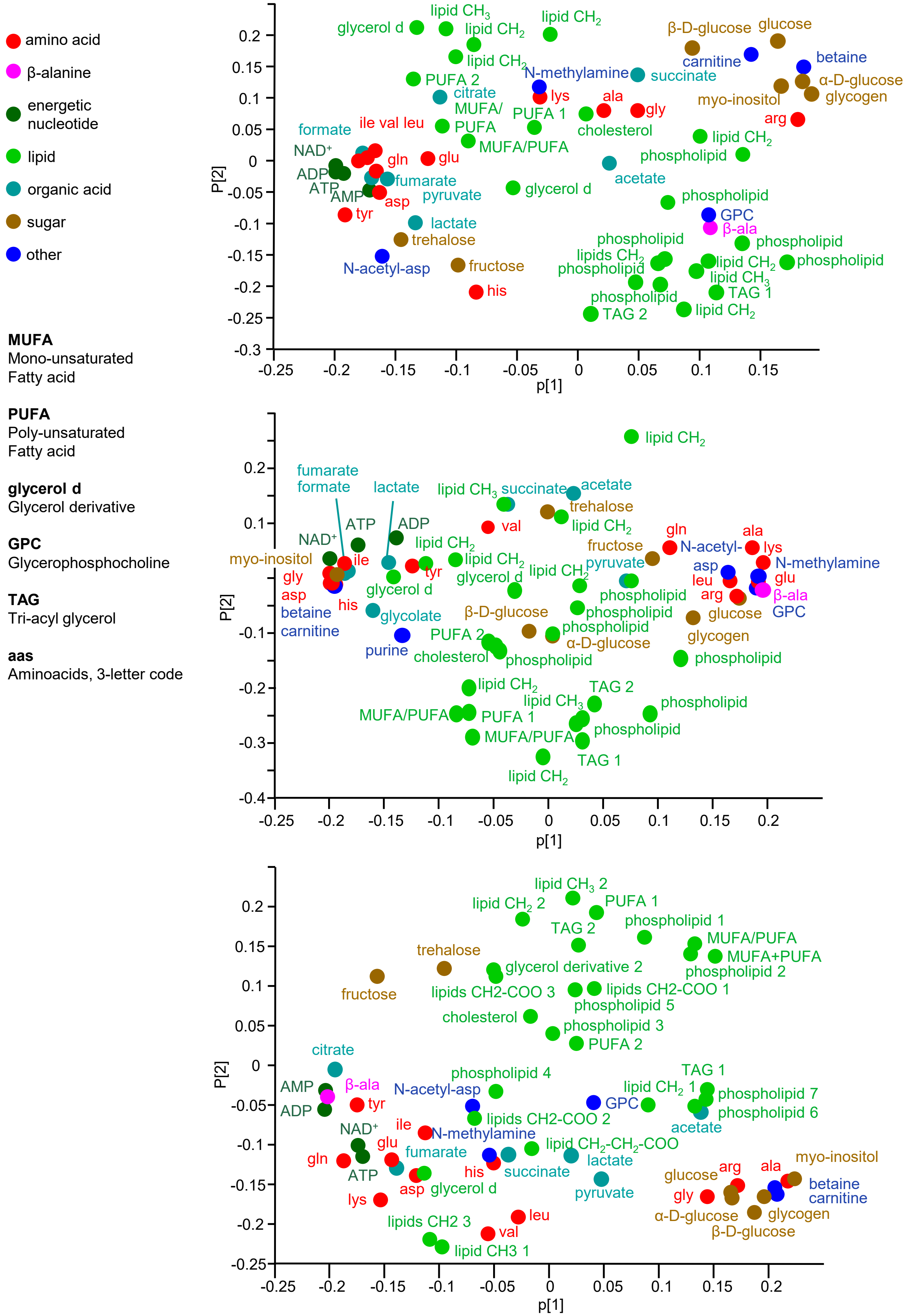


**Figure S1. Loading plots from Figure 2 with individual metabolite assignation.**

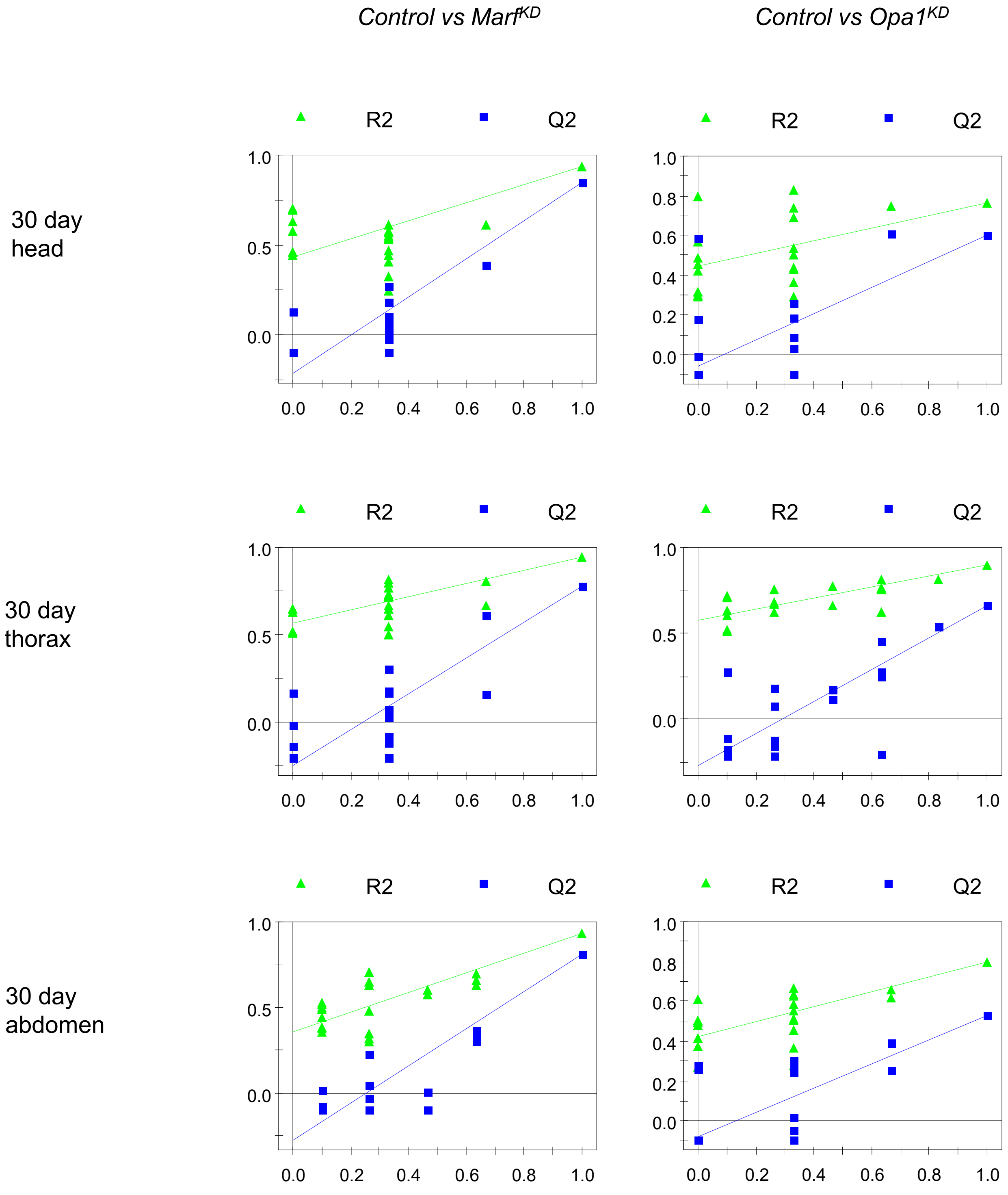


**Figure S2. OPLS by pairs at 30 days**. The anatomical regions of each one of the RNAi lines was compared to the control flies. In all six cases, a good model was found for the separation of the genotypes.

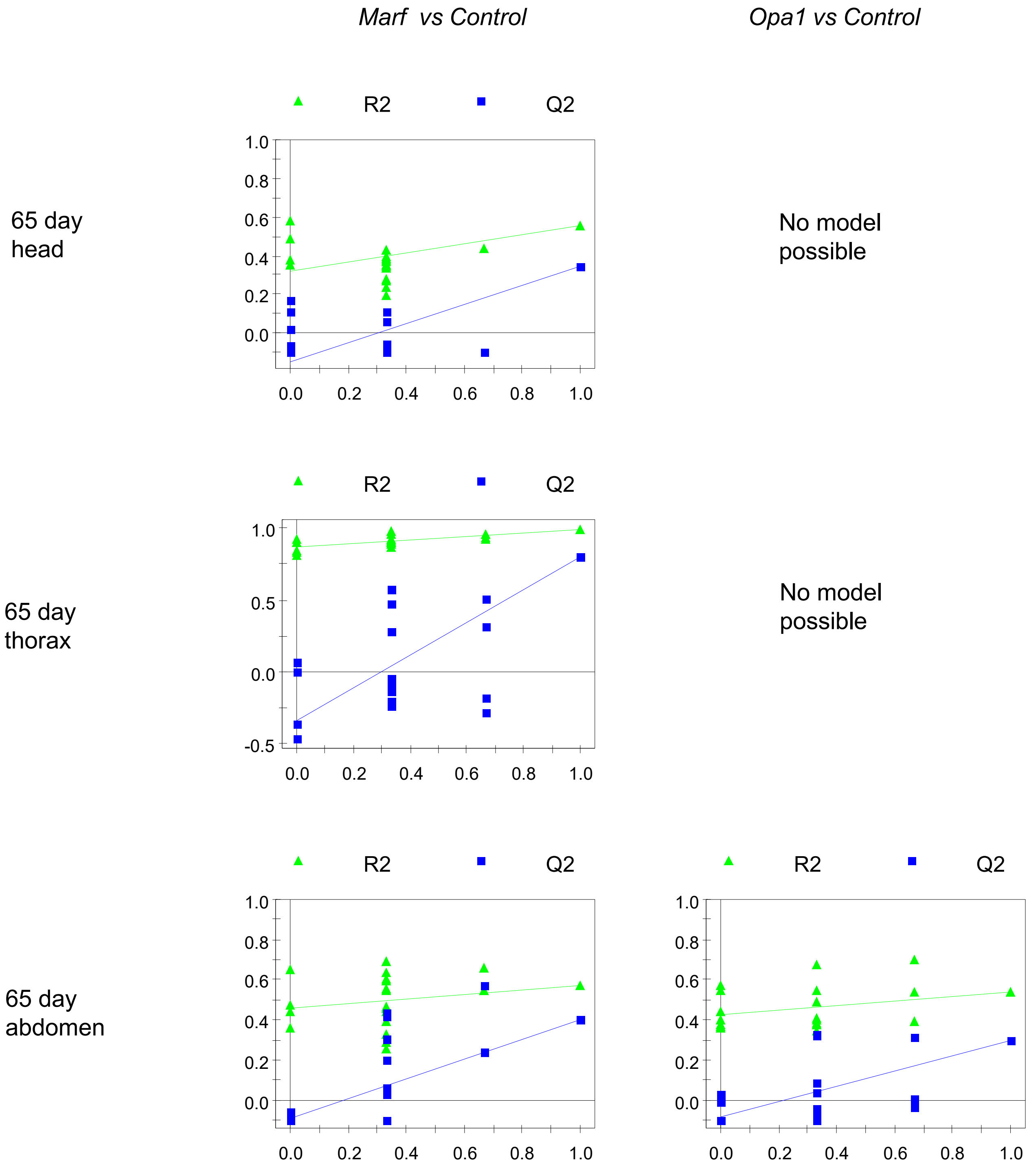


**Figure S3. OPLS by pairs at 65 days**. The anatomical regions of each one of the RNAi lines was compared to the control flies. In this case a model could be found only for four of the six comparisons, and they were less robust than at 30 days.

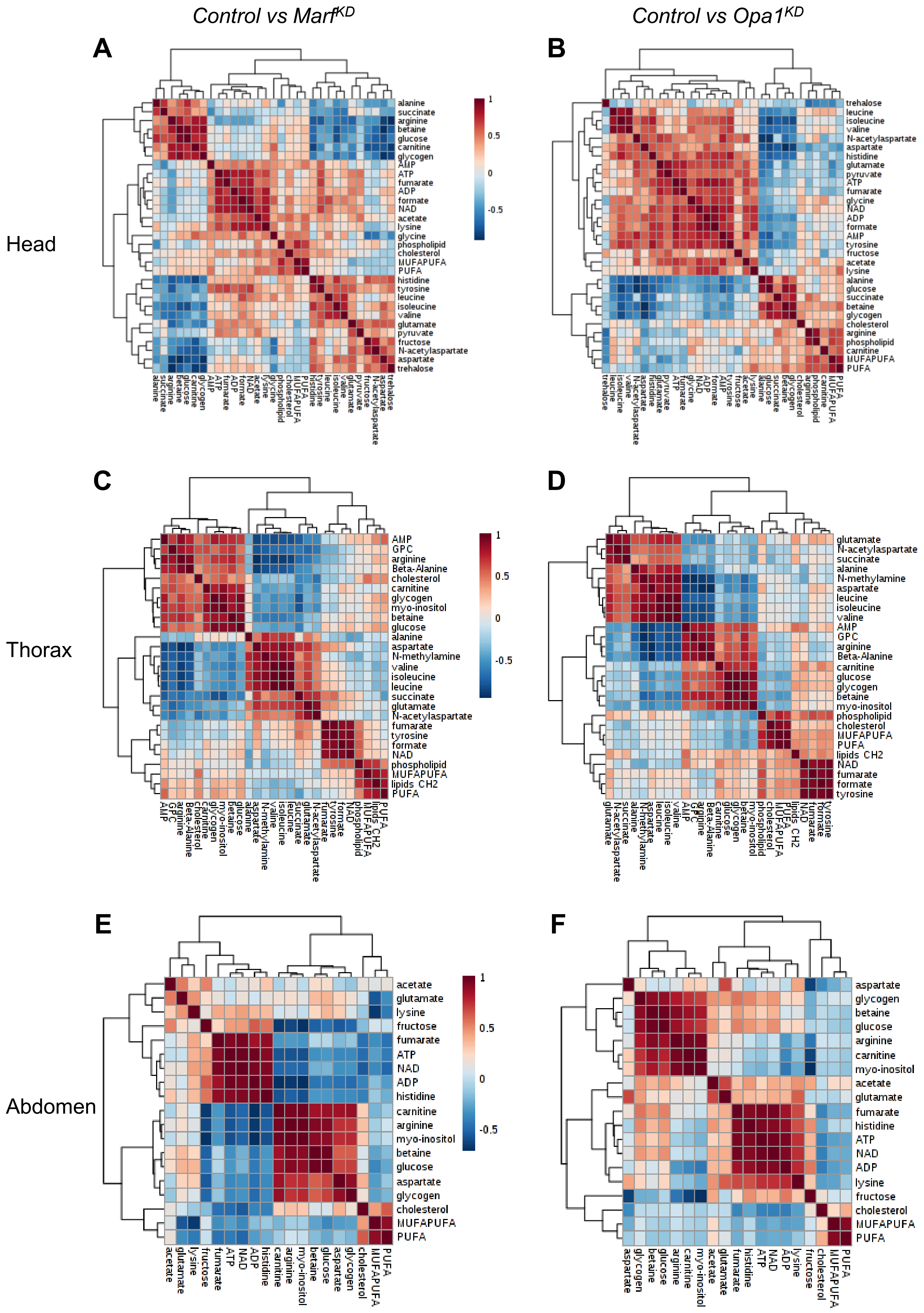


**Figure S4. Correlation matrixes of metabolites at 30 days**. **A**. Heads, *Control versus Marf*$^{KD}$. **B**. Heads, *Control versus Opa1*$^{KD}$. **C**. Thorax, *Control versus Marf*$^{KD}$. **D**. Thorax, *Control versus Opa1*$^{KD}$. **E**. Abdomen, *Control versus Marf*$^{KD}$. **F**. Abdomen, *Control versus Opa1*$^{KD}$.
